# Propagation of Rhinovirus C in Differentiated Immortalized Human Airway HBEC3-KT Epithelial Cells

**DOI:** 10.3390/v11030216

**Published:** 2019-03-04

**Authors:** Mina Nakauchi, Noriyo Nagata, Ikuyo Takayama, Shinji Saito, Hideyuki Kubo, Atsushi Kaida, Kunihiro Oba, Takato Odagiri, Tsutomu Kageyama

**Affiliations:** 1 Influenza Virus Research Center, National Institute of Infectious Diseases, 4-7-1 Gakuen, Musashimurayama-shi, Tokyo 208-0011, Japan; i-taka@nih.go.jp (I.T.); ssaito@nih.go.jp (S.S.); todagiri@nih.go.jp (T.O.); tkage@nih.go.jp (T.K.); 2 Department of Pathology, National Institute of Infectious Diseases, 4-7-1 Gakuen, Musashimurayama-shi, Tokyo 208-0011, Japan; nnagata@nih.go.jp; 3 Division of Microbiology, Osaka Institute of Public Health, 8-34 Tojo-cho, Tennoji-ku, Osaka 543-0026, Japan; hide-kubo@iph.osaka.jp (H.K.); a-kaida@iph.osaka.jp (A.K.); 4 Department of Pediatrics, Showa General Hospital, 8-1-1 Hanakoganei, Kodaira-shi, Tokyo 187-0002, Japan; oba.k@showa-hp.jp

**Keywords:** rhinovirus C, air–liquid–interface culture, immortalized cells, clinical isolates

## Abstract

Rhinoviruses (RVs) are classified into three species: RV-A, B, and C. Unlike RV-A and -B, RV-C cannot be propagated using standard cell culture systems. In order to isolate RV-Cs from clinical specimens and gain a better understanding of their biological properties and pathogenesis, we established air–liquid-interface (ALI) culture methods using HBEC3-KT and HSAEC1-KT immortalized human airway epithelial cells. HBEC3- and HSAEC1-ALI cultures morphologically resembled pseudostratified epithelia with cilia and goblet cells. Two fully sequenced clinical RV-C isolates, RV-C9 and -C53, were propagated in HBEC3-ALI cultures, and increases in viral RNA ranging from 1.71 log_10_ to 7.06 log_10_ copies were observed. However, this propagation did not occur in HSAEC1-ALI cultures. Using the HBEC3-ALI culture system, 11 clinical strains of RV-C were isolated from 23 clinical specimens, and of them, nine were passaged and re-propagated. The 11 clinical isolates were classified as RV-C2, -C6, -C9, -C12, -C18, -C23, -C40, and -C53 types according to their VP1 sequences. Our stable HBEC3-ALI culture system is the first cultivable cell model that supports the growth of multiple RV-C virus types from clinical specimens. Thus, the HBEC3-ALI culture system provides a cheap and easy-to-use alternative to existing cell models for isolating and investigating RV-Cs.

## 1. Introduction

Rhinoviruses (RVs) are positive-strand RNA viruses of the genus *Enterovirus* in the family *Picornaviridae*. The RV genome comprises a structural (VP4, VP2, VP3, and VP1) and non-structural (2A, 2B, 2C, 3A, 3B, 3C, and 3D) region flanked by 5′ and 3′ untranslated regions (UTRs) [1,2]. Recently, a genotypic classification of RVs based on the VP1 sequence was proposed. According to this classification, more than 160 types of RVs have been classified into three species (A, B, and C) (available online: http://www.picornaviridae.com/) [3]. Receptors of RV-A and -B are intercellular adhesion molecule 1 (ICAM-1) and low-density lipoprotein receptor (LDLR) family members [4,5], whereas those of RV-C comprise the recently identified human cadherin-related family member 3 (CDHR3), expressed in ciliated airway epithelial cells [6,7]. 

RVs cause respiratory illnesses worldwide. They constitute the most common causes of upper respiratory tract infections [8]. Next to respiratory syncytial viruses, RVs are the most common pathogens associated with severe bronchiolitis in children [9]. RV-A and -C are found predominantly in children with lower respiratory infections and wheezing illnesses. However, the differences in severity among these three RV species as well as the detailed role played by each RV species in respiratory diseases remain unclear [10,11,12,13]. 

Unlike RV-A and -B, RV-C cannot be propagated using standard cell cultures. To date, it has been reported that RV-C can be propagated in sinus organ culture, air–liquid–interface (ALI) cultured differentiated human airway epithelial cells, and commercially available three-dimensional (3D) human upper airway epithelia reconstituted in vitro [14,15,16,17]. These systems have provided an important tool for RV-C research. However, isolation of a sufficient number of sinus organs for culture and the stable culture of differentiated primary human airway epithelial cells are difficult. In addition, commercially available 3D human upper airway epithelial cells are costly, and only a few clinical isolates, RV-C2, -C7, -C12, -C15, and -C29, have been isolated using this method; of these, only RV-C7 and -C29 have been passaged and re-propagated [17]. Therefore, the isolation of RV-Cs from clinical specimens in order to gain a better understanding of their biological properties and pathogenesis requires stable and inexpensive culture systems.

In this study, we established the ALI culture method using immortalized human airway epithelial HBEC3-KT cells to isolate RV-C from clinical specimens. The morphology of the HBEC3-ALI culture resembled pseudostratified epithelia with cilia and goblet cells. Further, RV-C9 and -C53 propagated efficiently in HBEC3-ALI cultures, and 11 clinical RV-C strains were isolated from 23 clinical specimens.

## 2. Materials and Methods 

### 2.1. Cell Cultures

Human bronchial tracheal epithelial (HBTE) cells (FC-0035) were obtained from Lifeline Cell Technology (Carlsbad, CA, USA). HBEC3-KT (CRL-4051) and HSAEC1-KT (CRL-4050) cells were from the American Type Culture Collection (ATCC; Manassas, VA, USA). The cells were maintained in submerged cultures using PneumaCult-Ex medium (STEMCELL Technologies, Vancouver, Canada), according to the manufacturer’s instructions. HBTE, HBEC3-KT, and HSAEC1-KT cells were generated via ALI culture using Transwell Permeable Supports (Costar 3470, Corning Costar, Corning, NY, USA) and PneumaCult-ALI medium (STEMCELL Technologies), according to the manufacturer’s instructions. The cultures were allowed to mature for at least 28 days. MRC5 (CCL-171) cells were obtained from the ATCC and maintained in minimum essential medium containing 10% fetal calf serum. 

### 2.2. Histological Examination

HBEC3- and HSAEC1-ALI cells cultured for 30 days were fixed in 10% phosphate-buffered formalin and routinely embedded in paraffin. Paraffin-embedded sections were stained with hematoxylin and eosin, and periodic acid-Schiff staining was also performed.

### 2.3. Clinical Specimens and Viruses

A total of 23 nasopharyngeal swabs or nasal aspirates obtained from patients enrolled in clinical studies that were approved by the National Institute of Infectious Diseases, Showa General Hospital Ethics Committee, and which screened positive for RV by real-time RT-PCR and partial 5′UTR sequencing [18], were used for the study. 

RV-A16 (VR-283) was obtained from the ATCC and propagated using MRC5 cells. RV-C9 and -C53 were isolated from the clinical specimens using ALI-cultured HBTE cells according to previously described methods [15,16,19]. Whole genome sequences of these RV-C9 and -C53 strains were determined using a next-generation sequencer (see Section 2.9.2) and deposited at the National Center for Biotechnology Information (NCBI) (accession numbers LC428175 and LC428176).

### 2.4. Virus Inoculation

RV-C9, -C53 (8 and 7 log_10_ RNA copies/well), and RV-A16 (6 and 5 log_10_ RNA copies/well) were inoculated onto the apical surfaces of HBEC3- and HSAEC1-ALI cultures. After 4 h of incubation, the cells were washed three times with Opti-MEM (Thermo Fisher Scientific, Waltham, MA, USA) to remove residual virus inoculate and incubated at 34 °C. After incubation for 1, 3, 7, 14, and 21 days, the apical surfaces of the cells were washed with 200 μL of Opti-MEM to collect the propagated viruses. The viral genomes were then quantified by real-time RT-PCR. Culture media were also collected from the basolateral sides at 1, 7, 14, and 21 days following inoculation.

### 2.5. RNase Treatment 

Of the 200 μL of propagated viruses collected from the apical surface of HBEC3-ALI cultures 7 days after inoculation with 8 log_10_ RNA copies/well of RV-C9 and -C53, 6 μL was used for RNase treatment. A total 100 μL of reaction mixture containing 10 μL of 10× reaction buffer, 1 μL of RNase ONE™ Ribonuclease (10 U/μL; Promega, Madison, WI, USA), 83 μL of water, and 6 μL of sample was incubated for 30 min at 37 °C. To prepare the untreated samples, 1 μL of water was added to the reaction mixture instead of RNase ONE™ Ribonuclease.

### 2.6. RNA Extraction from Viruses

Total RNA was prepared from viral isolates using a MagMAX™ 96 Viral Isolation Kit (Thermo Fisher Scientific), using 50 μL of collected medium, with KingFisher Flex (Thermo Fisher Scientific) according to the manufacturer’s instructions. Total RNA was also prepared from 100 μL of RNase-treated samples using a MagMAX™ CORE Nucleic Acid Purification Kit (Thermo Fisher Scientific), using 60 μL of elution buffer, with KingFisher Flex (Thermo Fisher Scientific), according to the manufacturer’s instructions.

### 2.7. Amplification of the RV Genome by Real-Time RT-PCR 

Quantification of RV genomes was conducted via real-time RT-PCR using AgPath-ID™ One-Step RT-PCR Reagents (Thermo Fisher Scientific) as described previously [20], with a small modification. Briefly, the 10 μL assay contained 5 μL of 2× RT-PCR buffer, 0.4 μL of 25× RT-PCR Enzyme Mix, 1.4 μL of primers/probe mix, 0.2 μL of water, and 3 μL of template RNA. The primer/probe mix was prepared to contain 0.6 µM of each primer and 0.1 µM of the probe. To prepare the RNA transcript control, the 5′UTR region of RV-A16 was amplified by RT-PCR, and the resulting PCR product containing the T7 promoter was then transcribed in vitro. The 5′UTR region was amplified by PCR using Phusion High-Fidelity DNA Polymerase (New England BioLabs, Ipswich, MA, USA) with paired primers (TAATACGACTCACTATAGGGGTACWCTRKTAYTMYGGTAMYYTTGTACGCC and AGWGCATCKGGYAAYTTCCA). RNA was transcribed using the T7 RiboMAX™ Express Large-Scale RNA Production System (Promega) and treated with TURBO^®^ DNase to degrade the template DNA. The dNTPs and NTPs were removed using MicroSpin G-25 Columns (GE Healthcare, Piscataway, NJ, USA) according to the manufacturer’s instructions. The transcribed RNA was quantified using a NanoDrop spectrophotometer, and the absorbance value was used to calculate the copy numbers of the transcribed RNA. The integrity of the transcribed RNAs was assessed via a 2100 BioAnalyzer (Agilent Technologies, Santa Clara, CA, USA).

### 2.8. Virus Isolation

RV-positive specimens were inoculated onto the apical surfaces of HBEC3-ALI cultures. After 4 h of incubation, the cells were washed three times with Opti-MEM containing 10^2^ U/10^2^ μg/mL of penicillin/streptomycin, 100 μg/mL of gentamicin, and 0.5 μg/mL of amphotericin B to remove residual specimen inoculate, and the cultures were incubated at 34 °C. After incubation for 7, 14, and 21 days, the apical surfaces of the cells were washed with 200 μL of Opti-MEM to collect the propagated viruses.

### 2.9. Sequencing

#### 2.9.1. RT-PCR and Sanger Sequencing of VP1 to Type RVs

Purified viral RNA was reverse-transcribed using random hexamer primers (Promega) and Superscript III reverse transcriptase (Invitrogen, Carlsbad, CA) according to the manufacturer’s instructions. The cDNA (3 µL) was amplified in PCR using paired primers with Phusion High-Fidelity DNA Polymerase. The PCR cycling conditions were as follows: 98 °C for 30 s, 40 cycles at 98 °C for 10 s, 65 °C for 30 s, and 72 °C for 1 min, with a final extension of 72 °C for 10 min. The primer sequences for amplifying the VP1 region of each virus isolate are provided (Table S1). Amplified VP1 products of approximately 400 bp were then gel-purified with the MinElute Gel Extraction kit (Qiagen, Hilden, Germany), and sequencing was performed in both directions using amplification primers and the ABI Prism BigDye Terminator Cycle Sequencing Ready Reaction Kit, version 3.1.

#### 2.9.2. Next-Generation Sequencing

A next-generation sequencing (NGS) library was prepared using a NEBNext Ultra RNA Library Prep Kit (New England BioLabs), followed by the sequencing of 75-bp paired-end reads with MiSeq (Illumina, San Diego, CA, USA). The obtained sequencing reads were assembled using CLC Genomics Workbench 7 software (Qiagen) with De Novo Assembly.

### 2.10. Statistical Analyses

Statistical analyses were performed using the Prism statistical software package (version 7.0; GraphPad Software, Inc., La Jolla, CA, USA). One-way analysis of variance (ANOVA) followed by Tukey’s multiple comparisons test was used to analyze the growth properties of the RVs. Two-way ANOVA followed by Sidak’s multiple comparisons test was used to analyze the increased RNA levels of the RVs. A Welch’s unpaired t test was used to compare the RNA copy numbers of RV genomes among clinical specimens.

## 3. Results

### 3.1. RV-C9, -C53, and RV-A16 Growth in HBEC3- and HSAEC1-ALI Cultures

To obtain RV-C strains, RV-C9 and -C53 were isolated from the clinical specimens using ALI-cultured primary HBTE cells according to a previously established culture system for RV-Cs [15,16]. They were used as reference strains to evaluate our established culture system in this study. Whole-genome sequences of RV-C9 and -C53 were determined via NGS (accession numbers, LC428175 and LC428176).

**Figure 1:**
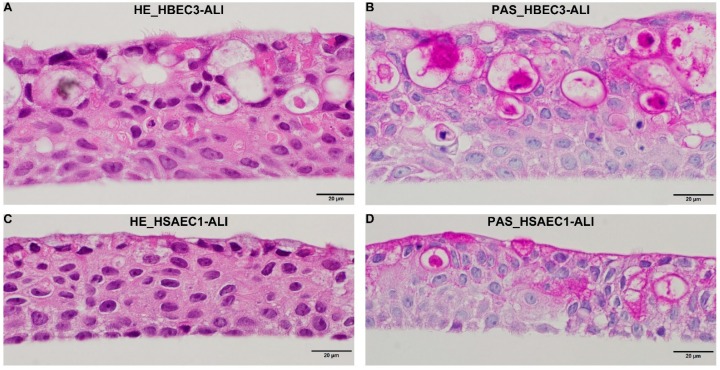
Histological morphology of HBEC3 and HSAEC1 cells cultured using the air–liquid–interface (ALI) system. Hematoxylin and eosin (HE) staining (**A**,**C**) and periodic acid-Schiff (PAS) staining (**B**,**D**) of HBEC3-ALI (**A**,**B**) and HSAEC1-ALI (**C**,**D**) cultures.

In order to establish a stable and inexpensive culture system for RV-Cs, HBEC3-KT and HSAEC1-KT cells were cultured under ALI conditions. After being maintained in the ALI system for 30 days, histological examination revealed that HBEC3- and HSAEC1-ALI cultures were likely pseudostratified human airway epithelial cells with cilia and mucus-secreting goblet-like cells (Figure 1). 

**Figure 2:**
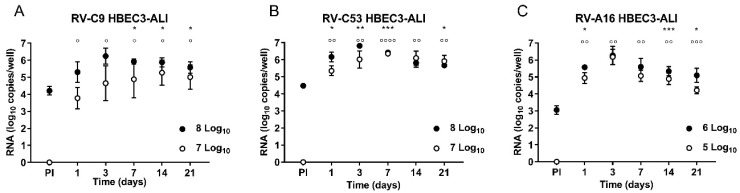
Growth properties of rhinoviruses (RVs) in HBEC3 air–liquid-interface (ALI) culture. For this, 8 or 6 (filled circles) and 7 or 5 (open circles) log_10_ RNA copies/well of RV-C9 and -C53 or RV-A16, respectively, were inoculated onto HBEC3-ALI cultures (**A**–**C**). Data are expressed as log copies of RNA per well (*n* = 3) at 1, 3, 7, 14, and 21 day(s) after inoculation. PI represents the collected third wash solution. Differences in log RNA copies were analyzed by one-way ANOVA followed by Tukey’s multiple comparisons test. Asterisks (*) and circles (°) show differences between PI and each time point within 8 log_10_ RNA copies/well of RV-Cs/6 log_10_ RNA copies/well of RV-A inoculated groups and within 7 log_10_ RNA copies/well of RV-Cs/5 log_10_ RNA copies/well of RV-A inoculated groups. */° *p* < 0.05, **/°° *p* < 0.01, ***/°°° *p* < 0.001, and ****/°°°° *p* < 0.0001.

**Figure 3:**
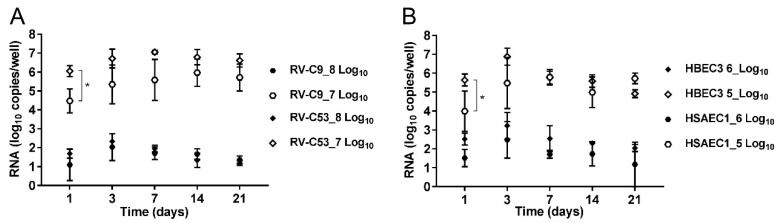
Increased RNA levels of RVs in HBEC3- and/or HSAEC1-ALI cultures. Data are expressed as log copies of RNA per well (*n* = 3) at 1, 3, 7, 14, and 21 day(s) post-inoculation and normalized to the RNA concentration of PI (collected third wash solution) as 0. (**A**) Comparison of increased RNA levels between RV-C9 and -C53 in HBEC3-ALI cultures. Groups comprising inoculums of 8 log_10_ RNA copies/well of RV-C9 (filled hexagons) and RV-C53 (filled diamonds), and 7 log_10_ RNA copies/well of RV-C9 (open hexagons) and RV-C53 (open diamonds) were compared at each time point. (**B**) Comparison of increased RV-A16 RNA levels between HSAEC1- and HBEC3-ALI cultures. Groups comprising inoculums of 6 log_10_ RNA copies/well of RV-A16 in HBEC3- (filled diamonds) and HSAEC1- (filled hexagons) ALI cultures and 5 log_10_ RNA copies/well of RV-A16 in HBEC3- (open diamonds) and HSAEC1- (open hexagons) ALI cultures were compared at each time point. Differences in log RNA copies were analyzed by two-way ANOVA followed by Sidak’s multiple comparisons test. * *p* < 0.05.

In order to observe the growth properties of the RVs in HBEC3- and HSAEC1-ALI cultures, RV-C9 and -C53 (8 and 7 log_10_ RNA copies/well), as well as RV-A16 (6 and 5 log_10_ RNA copies/well), were inoculated in three replicates for each RNA copy number of RV. Samples were collected from the apical surfaces at 1, 3, 7, 14, and 21 days following inoculation, and RNA levels were analyzed by comparing the amount of RNA in PI samples (the collected third wash solution). Following inoculation of HBEC3-ALI cultures with 8 and 7 log_10_ RNA copies/well of RV-C9, the RNA levels increased significantly from 4.21 (PI) to 5.92 log_10_ copies/well at day 7 and from under the detection limit (PI) to 3.78 log_10_ copies/well at day 1, respectively (Figure 2A). Following inoculation of HBEC3-ALI cultures with the same amounts of RV-C53 as that of RV-C9, the RNA levels increased significantly from 4.48 (PI) to 6.17 log_10_ copies/well and from under the detection limit (PI) to 5.35 log_10_ copies/well at day 1, respectively (Figure 2B). However, inoculation of HSAEC1-ALI cultures with similar amounts of RV-C9 and -C53 did not lead to significantly higher RNA levels on any of the days tested, and only a slight increase in RNA was observed at day 3 following inoculation with 8 log_10_ RNA copies/well of RV-C53 (Figure S1A,B). There was no difference at any time point between RV-C9 and -C53 in the amounts of RNA in HBEC3-ALI cultures following inoculation with 8 log_10_ RNA copies/well. Following inoculation with 7 log_10_ RNA copies/well, the amount of RV-C53 RNA was significantly higher at day 1 compared to that for RV-C9, but there were no differences at days 3, 7, 14, or 21 (Figure 3A). 

To compare the growth properties of RV-Cs and RV-A, HBEC3-ALI cultures were inoculated with 6 and 5 log_10_ RNA copies/well of RV-A16. At day 1, RNA levels increased significantly from 3.05 (PI) to 5.57 log_10_ copies/well and from under the detection limit (PI) to 4.94 log_10_ copies/well, respectively (Figure 2C). Contrary to what observed for RV-Cs, the RNA levels also increased significantly from under the detection limit (PI) to 5.09 log_10_ copies/well at day 7 following inoculation of HSAEC1-ALI cultures with 5 log_10_ RNA copies/well of RV-A16 (Figure S1C). There were no differences in the amounts of RV-A16 RNA between HBEC3- and HSAEC1-ALI cultures, except for day 1 following inoculation with 5 log_10_ RNA copies/well, when the RNA level was significantly higher in the HBEC3-ALI cultures (Figure 3B).

Both RV-A and -C were detected only on the apical surfaces and not in the basolateral cultured medium (data not shown), and no cytopathic effects were observed after inoculating HBEC3- or HSAEC1-ALI cultures with RV-A and -C (data not shown).

To confirm that an increased in RNA indicated encapsidated progeny RNA genomes, samples collected from the apical surfaces of HBEC3-ALI cultures at day 7 post-inoculation with 8 log_10_ RNA copies/well RV-C9 and -C53 were treated with RNase under conditions that the non-encapsidated viral RNA would be completely degraded. Comparison of the amounts of RNA between RNase-treated and untreated samples following RNA extraction by real-time RT-PCR indicated no significant difference (Figure S2).

### 3.2. Isolation of Clinical RV-C Isolates Using HBEC3-ALI Culture

**Figure 4:**
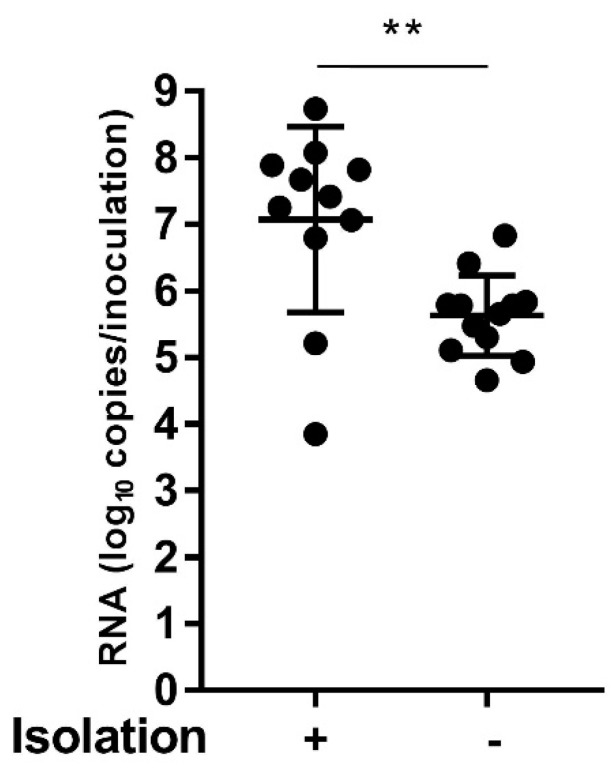
Comparison of RNA copy numbers of RV genomes between the 11 clinical specimens from which the viruses were successfully isolated and the 12 clinical specimens from which the viruses could not be isolated. Differences were analyzed by Welch’s unpaired t test. ** *p* < 0.01.

Twenty-three samples that were positive for RV-C by real-time RT-PCR and 5′UTR sequence analysis were inoculated onto the apical surface of HBEC3-ALI cultures, and 11 RV-C clinical isolates were successfully isolated (Table S2). A phylogenetic tree based on the VP1 sequences of these 11 RV-C clinical strains and on corresponding sequences of RV-C prototypes showed that they could be classified into eight types: RV-C2, -C6, -C9, -C12, -C18, -C23, -C40, and -C53 (Table S2 and Figure S3A). In addition, nine of the 11 clinical isolates including RV-C2, -C6, -C9, -C12, -C18, -C23, and -C53 collected from the apical surfaces were subsequently passaged and re-grown in HBEC3-ALI cultures (data not shown). In order to determine the potential causes for our failure to isolate RV-Cs from the 12 samples, we compared the contained RNA copy numbers of RV genome between the 11 clinical specimens from which the viruses were isolated successfully and the 12 clinical specimens from which the viruses could not be isolated. Consequently, it was evident that the contained RNA copy numbers in the specimens from which the viruses were successfully isolated were significantly higher (Figure 4). 

## 4. Discussion

Reportedly, RV-Cs target ciliated airway epithelial cells expressing the only known RV-C cell-entry factor, CDHR3 [7]. HBEC3-KT and HSAEC1-KT cells are normal human bronchial epithelial cells immortalized with CDK4 and hTERT that retain the capacity to differentiate into mucin-producing and ciliated epithelial cells in ALI culture using collagen gel [21,22,23]. In this study, HBEC3-KT and HSAEC1-KT cells were cultured under ALI conditions using Transwell Permeable Supports and PneumaCult ALI medium and differentiated into pseudostratified epithelia with cilia and goblet cells (Figure 1). Although no obvious morphological differences were observed between HBEC3- and HSAEC1-ALI cultures, RV-C9, -C53, and -A16 propagated well in HBEC3-ALI cultures (Figure 2A–C), whereas only RV-A16 propagated well in HSAEC1-ALI cultures (Figure S1A–C). Considering these results, the different RV-C growth properties between HBEC3- and HSAEC1-ALI cultures were not dependent on cell morphology, and therefore other unknown host cell factor(s) might be responsible for the differences. Further studies are needed to elucidate such discrepancies.

RNA amounts were not affected by the treatment of the collected samples with RNase before RNA extraction (Figure S2). This suggested that the increased RNA in HBEC3-ALI cultures indicated encapsidated progeny RNA genomes. The most significant increases in RNA levels in HBEC3-ALI cultures inoculated with 8 and 7 log_10_ RNA copies/well of RV-C9 were 1.71 and 5.97 log_10_ copies/well, respectively, and in HBEC3-ALI cultures inoculated with RV-C53 were 2.34 and 7.06 log_10_ copies/well, respectively (Figure 3A). These increases in RNA levels agree with those of previous reports on RV-C culture methods [15,16,17]. In the HBEC3-ALI cultures, after virus inoculation onto the apical surface, propagated viruses were detected only on the apical surfaces and not in basolateral cultured medium (data not shown). These observations also agree well with previous reports on RV-C culture methods [14,15,17] as well as with those on natural RV infections in human airway epithelia [24,25,26,27,28]. Considering that HBEC3-KT cells were stably cultured and recapitulated the pseudo-morphology of human airway epithelia with the ability to serve as hosts for sufficient RV-C propagation—as in natural RV infections—our established HBEC3-ALI culture appears to be a stable culture system suitable for investigating RV-Cs.

Using HBEC3-ALI cultures, 11 clinical strains were isolated from 23 clinical specimens. The mean RNA copy numbers of RV genome in the 11 clinical specimens from which the viruses were successfully isolated was 7.07 log_10_ copies/inoculation (Figure 4). Considering that RNA copy numbers in the 11 specimens from which the viruses were successfully isolated were significantly higher, it is surmised that we failed to isolate RV-Cs from the other 12 clinical specimens partly because they contained lower concentrations of RV-C. The clinical isolate 47-SGH-JPN-2015 was isolated from the same clinical specimen as that of RV-C9 using ALI-cultured HBTE cells. Similarly, 4-SGH-JPN-2015 was isolated from the same clinical specimen as that of RV-C53. It was shown that there were no differences between the VP1 sequences of RV-C9 and -C53, as determined by NGS (accession numbers, LC428175 and LC428176) and between those of 4-SGH-JPN-2015 and 47-SGH-JPN-2015, as determined by Sanger sequencing (accession numbers, LC428172 and LC428173). These results suggest that our established HBEC3-ALI culture did not affect the VP1 sequence. Moreover, it is speculated that the same strain might be isolated from a clinical specimen using either HBEC3-ALI culture or ALI-cultured HBTE cells.

In conclusion, we established a stable ALI culture system, the HBEC3-ALI culture, using immortalized human airway epithelial cells in which RV-C propagated well, and showed that 11 RV-C clinical strains could be isolated using this system. Further, nine of the 11 isolates were re-propagated in HBEC3-ALI cultures. Thus, the established culture system will ultimately help to gain an understanding not only of basic replication mechanisms but also of the clinical features of RVs under conditions that more closely mimic natural infections.

## Supplementary Materials

The following are available online at https://www.mdpi.com/1999-4915/11/3/216/s1, Table S1: Primers for sequencing the VP1 region. Table S2: Clinical isolates of RV-Cs. Figure S1: Growth properties of RVs in HSAEC1-ALI culture. Figure S2: Comparison of RNA amounts between RNase-treated (+) and -untreated (–) samples of RV-C9 and -C53 samples. Figure S3: Phylogenetic analysis of the VP1 genes of clinical isolates and prototypes of RV-C.
