# Supplementary material for: Propagation of Rhinovirus C in Differentiated Immortalized Human Airway HBEC3-KT Epithelial Cells
Source: Viruses. 2019 Mar 4;11(3):216. doi: 10.3390/v11030216 (PMC6466094; doi:10.3390/v11030216)
Supplement: Supplementary file 1 [file viruses-11-00216-s001.pdf]

viruses

MDPI

Supplementary Information

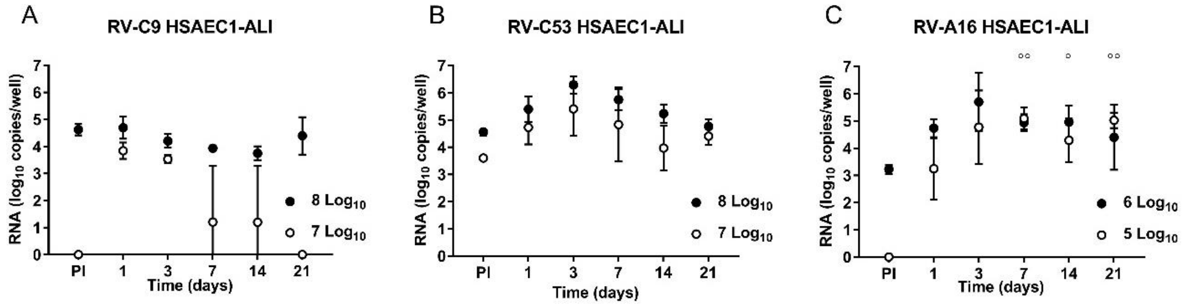

Figure S1. Growth properties of rhinoviruses (RVs) in HSAEC1 air–liquid–interface (ALI) culture. For this, 8 or 6 (filled circles) and 7 or 5 (open circles) $\log_{10}$ RNA copies/well of RV-C9 and -C53 or RV-A16, respectively, were inoculated onto HSAEC1-ALI cultures (A, B, and C). Data are expressed as log copies of RNA per well (n = 3) at 1, 3, 7, 14, and 21 day(s) after inoculation. PI represents the collected third wash solution. Differences in log RNA copies were analyzed by one-way ANOVA followed by Tukey's multiple comparisons test. Asterisks (*) and circles (°) show differences between PI and each time point within 8 $\log_{10}$ RNA copies/well of RV-Cs/6 $\log_{10}$ RNA copies/well of RV-A inoculated groups and within 7 $\log_{10}$ RNA copies/well of RV-Cs/5 $\log_{10}$ RNA copies/well of RV-A inoculated groups. */° $p < 0.05$, **/°° $p < 0.01$, ***/°°° $p < 0.001$, and ****/°°°° $p < 0.0001$.

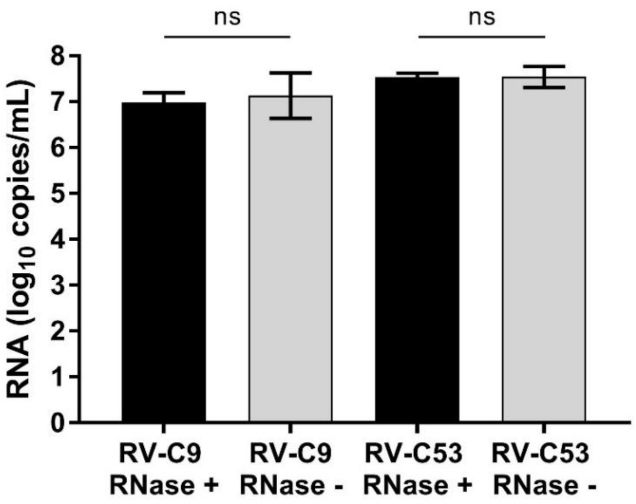

Figure S2. Comparison of RNA amounts between RNase-treated (+) and -untreated (–) RV-C9 and -C53 samples. Data are expressed as log copies of RNA per mL (n = 3). Differences in log RNA copies were analyzed by paired t tests for each RV. "ns" indicates not significant.

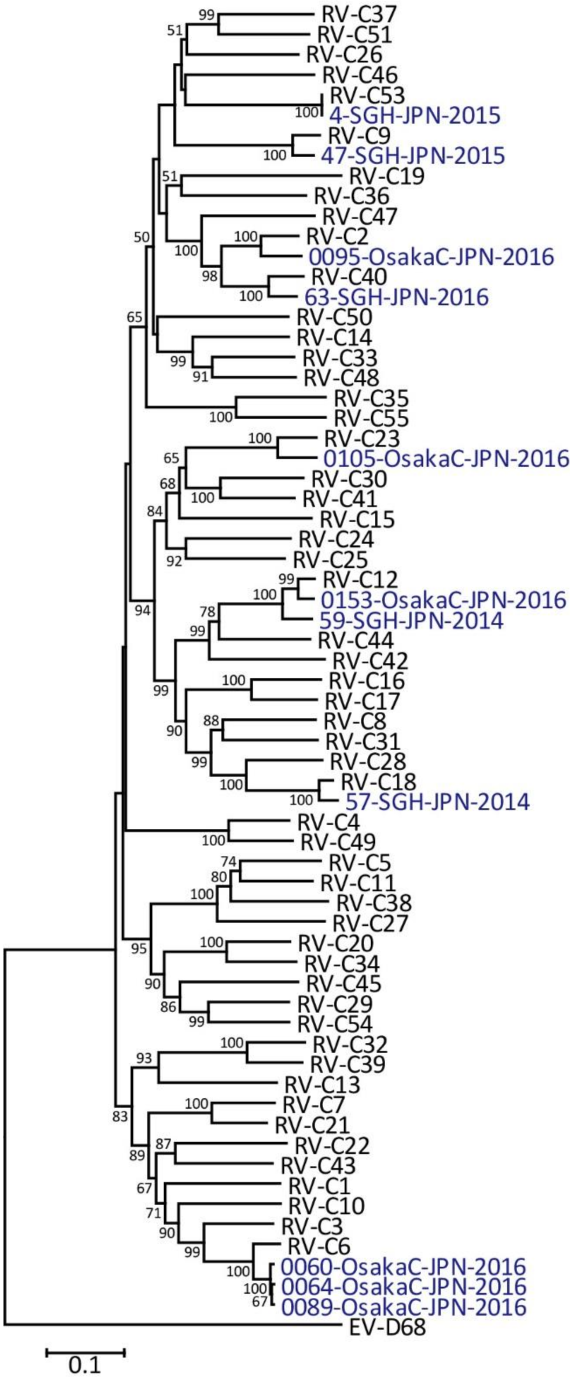

Figure S3. Phylogenetic analysis of the VP1 genes of the clinical isolates and prototypes of RV-C. The trees were constructed by a neighbor-joining method using the MEGA6 software and bootstrap values (>50%), calculated from 500 replicates and are shown to the left of the nodes. Evolutionary distances were computed using the Kimura 2-parameter method. Bars, 0.1 nt substitutions per site. VP1 sequences of clinical isolates were deposited at the National Center for Biotechnology Information (NCBI; accession numbers LC428164–LC428174). The sequence data of the prototypes were obtained from NCBI according to the proposal described previously [3] and the VP1 sequences of RV-C53 prototypes were obtained from NCBI (accession number MF775367). The VP1 sequence of Enterovirus D68 (EV D68) was also obtained from NCBI (accession number AY426531).

Table S1. Primers for sequencing the VP1 region.

Name	Sequence (5′ to 3′)	Virus isolate
HRVC02 VP1 F	GATACACCAATGATCAAACAACCTG	0095-OsakaC-JPN-2016
HRVC02 VP1 R	TTGTTCTGTGTGAACAAATAGGTCAC	
HRVC16-060 VP1 F	TCAAACAGCATTTGTATCCAATGGAACTAC	0060-OsakaC-JPN-2016
HRVC16-060 VP1 R	GTTAGGTTGATTGGGTAGTATCTGTCCCTG	
HRVC06 VP1 F	GACACACCTATGATGAGACAGGATG	0064-OsakaC-JPN-2016
HRVC06 VP1 R	GATGGCTTCCTTCGTGTGAAC	0089-OsakaC-JPN-2016
HRVC12 VP1 F	AAGAGACACACCCATGATGAAGC	0153-OsakaC-JPN-2016
HRVC12 VP1 R	GGTATAAATTGCATCCTTAGTATGGACATG	
HRVC14-59 VP1 F	GGTCAGGTAGTATAACCCTCACTTTCATG	59-SGH-JPN-2014
HRVC14-59 VP1 R	GCAACCTACTGTGCAGTCACATGTAGGTAT	
HRVC23 VP1 F	CCTGTCCAGACATGTCAGTTAGAATG	0105-OsakaC-JPN-2016
HRVC23 VP1 R	TGTTAAATGGGCGCACATGTACTTA	
HRVC40 VP1 F	AATGGCTAAACAACCTGACAACAATATC	63-SGH-JPN-2016
HRVC40 VP1 R	CTGTAGATGGCCTGTTGTGTGTGTAC	

Table S2. Clinical isolates of RV-Cs.

Virus isolate name	Pairwise nucleotide p-distances compared to prototype [a]	Type
0095-OsakaC-JPN-2016	0.092	C2
0060-OsakaC-JPN-2016	0.056	C6
0064-OsakaC-JPN-2016	0.057	C6
0089-OsakaC-JPN-2016	0.057	C6
47-SGH-JPN-2015	0.059	C9
0153-OsakaC-JPN-2016	0.040	C12
59-SGH-JPN-2014	0.064	C12
57-SGH-JPN-2014	0.040	C18
0105-OsakaC-JPN-2016	0.092	C23
63-SGH-JPN-2016	0.075	C40
4-SGH-JPN-2015	0.000	C53

[a] P-distances were calculated using the same sequence data of the prototypes used in Figure S3 with MEGA6 software.
